# Longitudinal Screening Detects Cognitive Stability and Behavioral Deterioration in ALS Patients

**DOI:** 10.1155/2018/5969137

**Published:** 2018-10-31

**Authors:** Susan Woolley, Ray Goetz, Pam Factor-Litvak, Jennifer Murphy, Jonathan Hupf, Catherine Lomen-Hoerth, Howard Andrews, Daragh Heitzman, Richard Bedlack, Jonathan Katz, Richard Barohn, Eric Sorenson, Bjorn Oskarsson, Americo Fernandes Filho, Edward Kasarskis, Tahseen Mozaffar, Sharon Nations, Andrea Swenson, Agnes Koczon-Jaremko, Georgia Christodoulou, Hiroshi Mitsumoto

**Affiliations:** ^1^Department of Neurosciences, Forbes Norris ALS Research Center, Sutter Pacific Medical Foundation, USA; ^2^Department of Psychiatry, New York State Psychiatric Institute, Columbia University Medical Center (CUMC), USA; ^3^Department of Epidemiology, CUMC, Mailman School of Public Health, USA; ^4^Department of Neurology, University of California, San Francisco (UCSF), USA; ^5^Department of Neurology, Eleanor and Lou Gehrig MD/ALS Research Center, CUMC, USA; ^6^Department of Neurology, UCSF, USA; ^7^Departments of Biostatistics and Psychiatry, CUMC, Mailman School of Medicine, USA; ^8^Texas Neurology, PA, USA; ^9^Duke University, USA; ^10^Department of Neurology, University of Kansas, USA; ^11^Mayo Clinic, Rochester, USA; ^12^University of California, Davis, Mayo Clinic, Jacksonville, USA; ^13^University of Nebraska Medical Center, USA; ^14^University of Kentucky, Lexington, USA; ^15^University of California, Irvine, USA; ^16^University of Texas, Southwestern, USA; ^17^University of Iowa, USA; ^18^Hospital for Special Care, USA

## Abstract

**Objective:**

To evaluate longitudinal cognitive/behavioral change over 12 months in participants enrolled in the ALS Multicenter Cohort Study of Oxidative Stress (ALS COSMOS).

**Methods:**

We analyzed data from 294 ALS participants, 134 of whom were studied serially. Change over time was evaluated controlling for age, sex, symptom duration, education, race, and ethnicity. Using multiple regression, we evaluated associations among decline in ALS Functional Rating Scale-Revised (ALSFRS-R) scores, forced vital capacity (FVC), and cognitive/behavioral changes. Change in cognitive/behavioral subgroups was assessed using one-way analyses of covariance.

**Results:**

Participants with follow-up data had fewer baseline behavior problems compared to patients without follow-up data. We found significant worsening of behavior (ALS Cognitive Behavioral Screen (ALS CBS) behavioral scale, *p* < 0.001; Frontal Behavioral Inventory-ALS (FBI-ALS) disinhibition subscale, *p* = 0.044). Item analysis suggested change in frustration tolerance, insight, mental rigidity, and interests (*p* < 0.05). Changes in ALSFRS-R correlated with the ALS CBS. Worsening disinhibition (FBI-ALS) did not correlate with ALSFRS-R, FVC, or disease duration.

**Conclusion:**

We did not detect cognitive change. Behavioral change was detected, and increased disinhibition was found among patients with abnormal baseline behavioral scores. Disinhibition changes did not correlate with disease duration or progression. Baseline behavioral problems were associated with advanced, rapidly progressive disease and study attrition.

## 1. Introduction

Patients with ALS may exhibit cognitive deficits, ranging from mild executive or language dysfunction to severe deficits consistent with dementia. For others, cognition may be relatively preserved yet behavioral symptoms are present, ranging from mild apathy to severe behavioral dysfunction reflecting frontotemporal dementia (FTD). ALS patients may demonstrate cognitive and behavioral deficits simultaneously, and those with marked deficits in both domains typically suffer from ALS-FTD.

ALS patients with concomitant FTD have shorter survival [1]. Nondemented patients with mild-moderate cognitive or behavioral abnormalities are also impacted prognostically. Patients who present with any degree of executive dysfunction at the time of diagnosis are more likely to exhibit faster cognitive and motor progression [2]. ALS patients who are cognitively normal at baseline are more likely to remain cognitively unchanged over time. Behavioral changes may predate both motor and cognitive symptoms in ALS [3], although less is known about longitudinal behavioral change in nondemented ALS patients. The recent study [4] found that severe apathy is a significant negative prognostic indicator in ALS regardless of severity of physical disease.

We previously reported that among participants enrolled in ALS COSMOS, rates of cognitive and behavioral impairment were consistent with existing literature, which supports the utility of cognitive and behavioral screening tools in large trials [5]. Here, we report on longitudinal data from the ALS COSMOS study in order to describe the natural history of both cognition and behavior in ALS.

## 2. Materials and Methods

### 2.1. Participant Selection and Consent

The ALS COSMOS study is a large, prospective, multicenter, interdisciplinary, epidemiological study of oxidative stress which has been described previously [6]. Two hundred ninety-four participants were enrolled at baseline. Current data is based on 134 participants with both baseline and follow-up assessments. The mean time interval between assessments was 11.5 ± 2.4 months.

IRB approval was obtained at each clinical site. Written informed consent was provided by all participants and their caregivers.

### 2.2. Cognitive and Behavioral Screening

Participants repeated a number of neuropsychological screens of cognitive and behavioral functioning. The ALS Cognitive Behavioral Screen (ALS-CBS) [7], the Verbal Fluency Index C words (VFI) [8], and Controlled Oral Word Association Test (COWAT) [9] were used to assess executive functioning, attention, verbal fluency, and mental control. The behavioral scale of the ALS CBS was used to evaluate for changes in personality, social comportment, and apathy based on caregiver's report. The Frontal Behavioral Inventory-ALS (FBI-ALS) version [10] was also used, which is an interview-based tool. The FBI-ALS is comprised of the negative scale (measures apathy, emotional flatness, and aspontaneity) and the disinhibition scale (measures behaviors like inappropriateness, impulsivity, and hyperorality). The Center for Neurological Study-Lability Scale (CNS-LS) [11], completed by the patient, is a screening tool for pseudobulbar affect (PBA). For further explanation of study measures, please refer to the baseline study [5].

### 2.3. Clinical and Demographic Variables

Functional disability was measured using the ALS Functional Rating Scale-Revised (ALS FRS-R) [12], and ventilatory status was measured using forced vital capacity (FVC). Duration of symptoms prior to diagnosis, time since diagnosis, and site of onset were recorded. Illness onset was defined as the first sign of muscle weakness and/or symptoms caused by muscle weakness [13]. Fasciculations, cramps, and fatigue were not considered signs of illness onset. The MMSE (Mini-Mental State Examination) [14] was administered at baseline but not at follow-up. Demographic variables included age, gender, education, and ethnicity. All data were stored in the Data Management Center at the Columbia University Medical Center [6].

### 2.4. Statistics

Statistical analyses were conducted using SPSS version 23.0 and SAS version 9.4. We identified a set of covariates that were associated with baseline ALS functional measures for all analyses: age, sex, duration of symptoms, education, race, and ethnicity. Analysis of covariance (ANCOVA) procedures were used to compare demographic and illness characteristics of cases with only baseline assessments versus cases with baseline and follow-up data. Visual inspection of histograms and Kolmogorov-Smirnov tests were used to assess the normality assumption of ANCOVA.

Follow-up cognitive assessments were performed 5–18 months after baseline (mean: 11.5 months). We calculated the time between the two assessments and categorized this time into quartiles. We examined all cognitive and behavioral measures by these quartiles and found no differences. Because there were no differences in cognitive and behavioral change related to the time between assessments, all cases were subsequently examined together.

Generalized estimating equations (GEE) were used to evaluate change over time for all measures, controlling for the a priori covariates. Multiple regression analysis was conducted to further examine changes over time. Change scores were calculated for cognitive, behavioral, and disease progression measures by comparing baseline scores to scores at follow-up. Negative change score values indicate a worsening of condition from baseline to 12-month follow-up. Change scores served as the dependent variable, while independent variables included the a priori covariates and the baseline score. Because the ALS CBS behavioral scores differed significantly from baseline to follow-up (decrement in function), we performed an item analysis of this scale first looking at the unadjusted changes in responses, then using general linear models, and controlling for covariates.

Using diagnostic criteria established by Strong et al. [15], we created diagnostic subgroups (normal, mild impairment, and moderate impairment) using the baseline ALS CBS cognition score, the baseline Verbal Fluency Index (normal and impaired) and the baseline ALS CBS behavioral score. Change scores were calculated for each diagnostic subgroup on each measure to examine the longitudinal progression of patients based on these classifications. We then performed a one-way ANCOVA to examine change scores across each diagnostic subgroup to determine whether baseline cognitive/behavioral diagnostic classification is associated with the degree of cognitive or behavioral change, if any. When significant differences were detected between diagnostic groups, we ran post hoc pairwise comparisons to determine specifically which groups differed from each other.

All tests were two-tailed, and significance was set at *p* < 0.05 for analyses. We performed sensitivity analyses using Bonferroni-corrected alpha levels to control for multiple comparisons.

## 3. Results

### 3.1. Comparison of Cases with and without Cognitive/Behavioral Follow-Up Data

Cases with follow-up cognitive/behavioral test scores had higher baseline ALSFRS-R (*p* < 0.001) and FVC (*p* < 0.001) ratings (Table 1), compared to cases without follow-up data. Cases in the follow-up group also had longer disease durations at baseline (*p* = 0.012) and were two years younger than those without follow-up (*p* = 0.047), although this age difference was no longer significant after Bonferroni correction. Other demographic variables did not differ significantly between groups.

Further, cases with follow-up data had significantly higher scores (reflecting fewer behavior problems) on the baseline ALS-CBS behavioral scale total compared to cases with no follow-up data (*p* = 0.036). No differences were found between cases with and without follow-up data on the other cognitive and behavioral tests (Table 1).

### 3.2. Cognitive Impairment at Follow-Up

Based on the ALS CBS cognitive score at follow-up, 5.2% of participants were classified in the range consistent with dementia and 54% in the mild cognitive impairment range (ALSci). This is consistent with baseline rates of impairment from the larger cohort (6.5% and 54.2%, respectively) [5]. Using the Verbal Fluency Index, 43.4% of the participants scored in the mildly impaired range at follow-up.

The cognitive screening tools (ALS CBS cognitive scale and VFI) did not reveal significant change during the follow-up period (Table 2). Although the sample means on the ALS CBS cognitive scale did not change over time (15.6 versus 15.4), regression analysis revealed that greater decline in performance was associated with being male (*p* = 0.034), longer duration between assessments (*p* = 0.033), and higher baseline CBS scores (*p* < 0.001). For sensitivity, we compared these *p* values to the Bonferroni-corrected alpha level of 0.005, for the 10 variables in the model. Higher baseline CBS scores persisted as a significant predictor of ALS CBS cognitive scale change using this conservative method. VFI decline was significantly associated with greater disease progression, as assessed by ALSFRS-R (*p* = 0.010), although fewer participants completed this screen at follow-up. The ALS CBS cognitive scale and COWAT did not associate with ALSFRS-R scores.

### 3.3. Behavioral Impairment at Follow-Up

Using ALS CBS behavioral cutoff scores [3], 14.9% of the sample scored within the range that suggests dementia at follow-up. This is equivalent with the percentage of patients in the baseline cohort with probable dementia at baseline (16.5%) [5]. Approximately 11.2% of participants scored within the range of mild behavioral impairment (ALSbi) at follow-up, comparable to 14.1% who were mildly impaired at baseline.

The ALS CBS behavioral total (*p* < 0.001) and the FBI-ALS disinhibition scale (*p* = 0.044) changed significantly during the follow-up period, suggesting behavioral deterioration, although the change in FBI-ALS disinhibition scale is no longer significant after Bonferroni correction (Table 2). Regression analysis showed that the decline in CBS behavior score was associated with disease progression (ALSFRS-R, *p* = 0.001). FBI disinhibition scores were not associated with any of the independent variables. The FBI-ALS negative scale remained stable from baseline to follow-up. Although the CNS-Lability Scale (CNS-LS) remained stable from baseline to follow-up, change on this measure was associated with disease progression (ALSFRS-R, *p* = 0.006 and FVC, *p* = 0.040) and the CNS-LS baseline score (*p* = 0.001).

We examined each item of the ALS CBS behavioral scale for change during follow-up. Without controlling for covariates, specific item scores that changed across the interval, reflecting deterioration in behavior, included the following: decreased interest in topics/events that used to be important, decreased ability to deal with frustration or stress, difficulty changing opinions/adapting to new situations, and decreased awareness of obvious problems/changes or denial. After adjusting for the same a priori covariates, all of these items except for “decreased interest in topics/events that used to be important,” were significantly different with *p* < 0.0001.

### 3.4. Change across Diagnostic Subgroups

Participants were classified into diagnostic subgroups (no impairment, mild impairment, and suspected dementia) according to consensus criteria [15] based on baseline ALS CBS scores. The ALS CBS cognitive change score differed among the three cognitive diagnostic subgroups (Table 3, *p* = <0.001). Participants in the no impairment and mild cognitive impairment subgroups had relatively stable cognitive scores between baseline and follow-up. Surprisingly, the dementia subgroup showed improvement on the CBS cognitive total (Table 3). No changes on other cognitive measures were found among baseline diagnostic subgroups.

Similar analyses were completed across behavioral diagnostic subgroups based on the baseline ALS CBS behavioral score. We detected significant decline (*p* = 0.008) between baseline and follow-up on the FBI disinhibition scale for all three behavioral diagnostic subgroups (Table 4). The greatest decline in disinhibition was found among participants classified in the dementia subgroup at baseline compared to the nonimpaired and participants with mild behavioral impairment (*p* = 0.002 and 0.056, respectively).

There were 6 cases identified as normal at baseline who transitioned to dementia at follow-up, exhibiting an expected and significant decline in behavioral scores (*p* = 0.002). However, these specific cases did not demonstrate an equivalent decline in ALS CBS cognitive scores (*p* = 0.320).

### 3.5. Site of ALS Onset

We examined all of the change scores with respect to the site of ALS onset (bulbar versus spinal), controlling for the a priori covariates. Bulbar-onset participants exhibited a greater decline in VFI (*p* = 0.013) compared to spinal-onset participants. No other change was evident.

## 4. Discussion

Significant behavioral change occurs in ALS patients over time, as indicated on screening measures completed by caregivers, including the FBI-ALS and ALS CBS. Behavioral change was not associated with disease duration or respiratory decline, but changes detected with the ALS CBS were associated with functional disability (ALSFRS-R scores). In contrast, disinhibition measured by the FBI-ALS was not associated with any disease severity indices.

Participants with behavioral problems consistent with FTD at baseline demonstrated more rapid progression of behavioral disinhibition over time compared to behaviorally normal participants or those with mild behavioral abnormalities. Despite the high prevalence of apathy in ALS [16, 17], significant change in this behavioral symptom was not detected using the FBI negative scale. However, an item analysis of the ALS CBS suggested progression of apathy, identified by change on an item measuring interest in topics and activities. Other behavioral symptoms that changed significantly over time included reduced frustration tolerance, reduced adaptability to new situations/changing opinions, and decreased insight. These results contribute to the existing literature that suggests that subtle behavioral changes are pervasive in ALS [3] and may be seen in patients without a clinically obvious syndrome [18].

Behavioral data was obtained from caregivers via a written questionnaire (ALS CBS) and interview (FBI-ALS). Caregiver perspectives can be biased by factors such as disease severity, mood, and psychosocial stress, which may lead to measurement error. The recent study found that behavioral impairment, but not cognitive impairment, negatively impacted caregiver burden in ALS [19]. Future behavioral studies may be strengthened by controlling for caregiver burden and caregiver depression/anxiety [20], which may provide an alternative explanation for increased rates of reported behavioral abnormalities over time and correlation with functional disability.

Using ALS CBS behavioral score cutoffs [7], nearly 15% of the sample fell within the range suggestive of FTD at follow-up. When FTD was estimated based on cognition rather than behavior, the rate was only 5.2%. It is possible that the rate discrepancy reflects the behavioral basis of FTD, since initial FTD symptoms frequently include social deficits or personality change rather than overt problems with concentration, set shifting, or fluency. Cognitive screening, if used without behavioral data, may underestimate the rate of dementia in ALS research cohorts.

Cognitive decline was not detected in this study. Study attrition correlates with cognitive impairment in ALS [2], and longitudinal studies may be enriched with cognitively intact patients [2]. Our results did not replicate this association, but we did detect an association between behavioral impairment and attrition. Participants who did not remain in the study were older and had more advanced disease that progressed more rapidly. These participants also had more baseline behavioral problems, suggesting an association between behavioral symptoms and more aggressive motor disease. Caga et al. [4] studied apathy and found that participants with the most severe behavioral impairment have significantly shorter mean survival (22 months) compared to those with mild apathy (46.9 months) or those without apathy (52 months).

Analysis of the baseline data from this cohort [5] suggested a significant correlation between apathy and cognitive impairment. These data also suggested a correlation between the absence of cognitive impairment and the presence of behavioral disinhibition. When considered within the context of attrition and longitudinal follow-up, the behavioral data makes sense. If longitudinal trials enrich for cognitively intact participants [2], then we may expect to see more disinhibition over time since it correlates with a relative lack of cognitive impairment. In contrast, since apathy correlated with cognitive impairment at baseline, we do not see marked increases in apathy over time because cognitively impaired patients may have a greater risk of attrition. These data suggest the possibility of two distinct phenotypes for further study: (1) patients with mild cognitive impairment and apathy and (2) cognitively normal patients with disinhibition.

## Figures and Tables

**Table 1:** Comparative analysis of baseline data for two groups: 134 cases with follow-up data compared to 160 cases without follow-up data.

Variable	Participants completing baseline and follow-up assessments (mean/SD)	*N*	Participants completing baseline assessment only (mean/SD)	*N*	*p*
Age	59.7 (10.1)	134	61.8 (9.9)	160	**0.076**
Onset to baseline assessment	14.3 (4.9)	134	12.8 (4.9)	160	**0.012**
Time from illness to enrollment	12.6 (4.4)	134	11.1 (4.3)	160	**0.005**
ALSFRS-R	38.5 (5.0)	129	34.2 (7.1)	154	**<0.001**
FVC	86.6 (20.5)	129	73.3 (22.4)	140	**<0.001**
% male	59.3	134	59	160	0.958
Race, % white	88.2	134	91.0	160	0.427
Hispanic ethnicity	9.3	134	4.5	160	0.108
Education		134		160	
% HS/GED	26.7%		20.9%		
Some college, AA	34.8%		29.1%		
BA, BS, or higher	38.5%		50.0%		0.138
MMSE	29.1 (1.4)	120	28.8 (2.0)	132	0.121
CBS cognitive score	15.6 (2.8)	120	15.1 (3.3)	134	0.126
CBS behavioral score	37.4 (5.2)	118	34.7 (6.9)	131	**0.036**
FBI negative scale	3.2 (4.9)	119	4.0 (4.7)	132	0.119
FBI disinhibition scale	1.45 (2.56)	119	1.87 (2.31)	132	0.127
CNS-Lability Scale	12.2 (4.5)	130	13.7 (5.4)	158	0.009
COWAT total	36.7 (10.7)	64	33.3 (11.6)	80	0.076
VFI C words	13.8 (7.7)	77	11.3 (6.9)	52	0.177

**Table 2:** Change over time: comparison of baseline and follow-up scores in 134 participants with longitudinal data, using GLM-GEE procedures, controlling for age, sex, duration of symptoms, education, race, and ethnicity (adjusted means and standard errors).

	Baseline mean (SE)	Follow-up mean (SE)	Fixed effects for interval	
			Wald chi-square value	*p* value
ALSFRS	38.5 (39.1)	27.6 (39.1)	240.27	**<0.001** ^⊥^
FVC%	86.7 (95.0)	64.5 (94.7)	114.13	**<0.001** ^⊥^
ALS CBS cognitive score	15.6 (9.8)	15.4 (9.8)	0.82	0.450
ALS CBS behavioral score	37.5 (31.0)	35.5^∗^ (30.9)	15.16	**<0.001** ^⊥^
Verbal Fluency Index (*z*-score converted)	−0.159 (3.79)	0.063^∗∗^ (3.74)	1.51	0.252
FBI negative scale	3.2 (20.3)	4.1 (20.2)	2.63	0.090
FBI disinhibition	1.46 (13.3)	2.01 (13.2)	4.07	**0.044**
CNS lability score	12.2 (20.2)	11.9^∗∗∗^ (20.2)	0.78	0.378

^∗^Decline in CBS behavioral score correlates with ALSFRS-R (*p* = 0.001). ^∗∗^Decline in VFI score correlates with ALSFRS-R (*p* = 0.010). ^∗∗∗^Decline in CNS-LS score correlates with ALSFRS-R (*p* = 0.006) and FVC (*p* = 0.040). ^⊥^Significant at the Bonferroni-adjusted alpha level of 0.007.

**Table 3:** Change in scores over time across diagnostic groups, defined by baseline ALS-CBS cognitive scores, controlling for age, sex, time from illness onset to baseline testing, and time between assessments (GLM ANCOVA, adjusted means, and standard errors are presented).

Cognitive and behavioral screens	Change scores across diagnostic subgroups (cognitive)^∗^						ANCOVA		Pairwise
	1 Normal		2 Mild		3 Moderate				
	*N* = 48–49		*N* = 60–62		*N* = 7–9				
	MN	SE	MN	SE	MN	SE	*F*	*p*	*p* < 0.05
ALS-CBS cognition	−1.12	0.373	0.13	0.335	4.33	0.983	13.92	0.001^∗^^⊥^	All
ALS-CBS behavior	−1.21	0.820	−2.50	0.730	−3.12	1.88	0.85	0.430	None
FBI negative	−0.89	0.82	−0.92	0.73	−.61	1.90	0.01	0.989	None
FBI disinhibition	−0.32	0.39	−0.77	0.35	−2.48	0.90	2.45	0.091	1 vs. 3
CNS-Lability Scale	−0.27	0.56	0.66	0.50	0.67	1.30	0.78	0.460	None

^∗^Diagnostic subgroups were determined based on baseline ALS CBS scores on the cognitive screen [7]. Normal: no significant cognitive impairment; mild: suspicion of mild cognitive impairment; moderate: suspicion of moderate-severe cognitive impairment, raising suspicion for dementia. ^⊥^Significant at the Bonferroni-adjusted alpha level of 0.01.

**Table 4:** Change in scores over time across diagnostic groups, defined by baseline ALS-CBS behavioral scores, controlling for age, sex, time from illness onset to baseline testing, and time between assessments (GLM ANCOVA, adjusted means, and standard errors are presented).

Cognitive and behavioral screens	Change scores across diagnostic subgroups (behavioral)^∗^						ANCOVA		Pairwise
	1 Normal		2 Mild		3 Moderate				
	*N* = 86–93		*N* = 10–11		*N* = 13–16				
	MN	SE	MN	SE	MN	SE	*F*	*p*	*p* < 0.050
ALS-CBS cognition	−0.23	0.313	−0.31	0.917	0.46	0.823	0.32	0.729	None
ALS-CBS behavior	−2.21	0.60	−3.00	1.71	−0.26	1.47	0.93	0.400	None
FBI negative	−0.79	0.59	−0.17	1.71	−1.96	1.52	0.35	0.708	None
FBI disinhibition	−0.37	0.274	−0.76	0.793	−2.80	0.706	5.01	0.008^∗^^⊥^	1 vs. 3
CNS-Lability Scale	0.46	0.42	−0.55	1.18	−0.22	1.02	0.45	0.640	None

^∗^Diagnostic subgroups were determined based on baseline ALS CBS scores on the behavioral screen [7]. Normal: no significant behavioral impairment; mild: suspicion of mild behavioral impairment; moderate: suspicion of moderate-severe behavioral impairment, raising suspicion for dementia. ^⊥^Significant at Bonferroni-adjusted alpha level of 0.01.

## Data Availability

This multicenter trial does not currently have IRB permission to share data. Permission was requested by the lead authors but the IRB had not granted approval by the time of publication.
