# Corrigendum to “Longitudinal Screening Detects Cognitive Stability and Behavioral Deterioration in ALS Patients”

**DOI:** 10.1155/2019/6704740

**Published:** 2019-12-04

**Authors:** Susan Woolley, Ray Goetz, Pam Factor-Litvak, Jennifer Murphy, Jonathan Hupf, Diana C. Garofalo, Catherine Lomen-Hoerth, Howard Andrews, Daragh Heitzman, Richard Bedlack, Jonathan Katz, Richard Barohn, Eric Sorenson, Bjorn Oskarsson, Americo Fernandes Filho, Edward Kasarskis, Tahseen Mozaffar, Sharon Nations, Andrea Swenson, Agnes Koczon-Jaremko, Georgia Christodoulou, Hiroshi Mitsumoto

**Affiliations:** ^1^Department of Neurosciences, Forbes Norris ALS Research Center, Sutter Pacific Medical Foundation, USA; ^2^Department of Psychiatry, New York State Psychiatric Institute, Columbia University Medical Center (CUMC), USA; ^3^Department of Epidemiology, CUMC, Mailman School of Public Health, USA; ^4^Department of Neurology, University of California, San Francisco (UCSF), USA; ^5^Department of Neurology, Eleanor and Lou Gehrig MD/ALS Research Center, CUMC, USA; ^6^Department of Neurology, UCSF, USA; ^7^Departments of Biostatistics and Psychiatry, CUMC, Mailman School of Medicine, USA; ^8^Texas Neurology, PA, USA; ^9^Duke University, USA; ^10^Department of Neurology, University of Kansas, USA; ^11^Mayo Clinic, Rochester, USA; ^12^University of California, Davis, Mayo Clinic, Jacksonville, USA; ^13^University of Nebraska Medical Center, USA; ^14^University of Kentucky, Lexington, USA; ^15^University of California, Irvine, USA; ^16^University of Texas, Southwestern, USA; ^17^University of Iowa, USA; ^18^Hospital for Special Care, USA

In the article titled “Longitudinal Screening Detects Cognitive Stability and Behavioral Deterioration in ALS Patients” [1], Dr. Diana C. Garofalo was missing from the authors' list. She reanalyzed all the primary and secondary outcomes, interpreted the new findings, and rewrote the stats section of the paper. The corrected authors' list is shown above.
